# Microcavity optomechanical magnetometry with picotesla-sensitivity

**DOI:** 10.1038/s41377-025-01767-4

**Published:** 2025-02-20

**Authors:** Zhaoyu Cai, Chengying Bao

**Affiliations:** State Key Laboratory of Precision Measurement Technology and Instruments, Department of Precision Instruments, Tsinghua University, Beijing, 100084 China

**Keywords:** Integrated optics, Imaging and sensing

## Abstract

A new microcavity magnetometry with FeGaB thin film achieves 1.68 pT/Hz^1/2^ sensitivity, which is two orders of magnitude improvement over previous work. Corona current detection has been demonstrated using this magnetometer.

High-quality (*Q*) whispering gallery mode (WGM) optical microcavities^1^ have emerged as a versatile platform for various applications, including quantum optics^2^, optical microcombs^3^, and highly sensitive sensing^4,5^. The eigenmodes of WGM microcavities are close to the waveguide surface; thus, the evanescent field is relatively strong and has been harnessed for sensitive detection of single proteins and viruses^4,5^. Moreover, the unique dual-resonance nature of optomechanical WGM microcavities, such as silica microtoroids, has unlocked new frontiers in quantum optomechanics^6^. For instance, these devices have facilitated the realization of quantum-coherent coupling^7^, optomechanically induced transparency^8^, and optomechanical cooling^9^. Beyond quantum phenomena, WGM optomechanical technologies have accelerated the development of state-of-the-art sensors, achieving unprecedented sensitivities in the detection of force^10^, acceleration^11^, and ultrasound^12,13^. Moreover, the integration of WGM microcavities with magnetostrictive materials has opened the door for a new class of microcavity optomechanical magnetometers^14,15^. Different from the state-of-the-art magnetometers such as superconducting quantum interference devices magnetometers^16^ and spin-exchange-relaxation-free atomic magnetometers^17^, microcavity optomechanical magnetometers offer advantages of room-temperature operation, chip-scale integration, and low power consumption.

Microcavity optomechanical magnetometers leverage the coupling between magnetic fields and mechanical motion to achieve highly sensitive optical detection of magnetic fields. When a magnetic field is applied, it induces strain in the magnetostrictive material, causing the microcavity to deform and alter its optical resonance frequency. When the frequency of the magnetic field matches the mechanical resonance frequency, the magnetic field induced force can significantly modulate the intracavity field. Due to the dual-enhancement of optomechanical response in high-Q WGM microcavities^6^, these magnetometers can reach extremely high sensitivity. There are two predominant structures for these microcavity magnetometers reported to date. The first approach involves manually embedding Terfenol-D particles within a microtoroid cavity^16^, enabling a sensitivity of 26 pT Hz^−1/2^. However, this technique is challenging for scaling. The second approach is to sputter coat Terfenol-D thin films within optical microcavities, which has better scalability^17^. Nevertheless, the sensitivity of these devices is limited to 585 pT Hz^−1/2^.

In a recent work published in *Light Science & Applications*, a research team led by Professor Bei-Bei Li from Institute of Physics, Chinese Academy of Sciences, has reported a highly sensitive and mass-producible microcavity optomechanical magnetometer (Fig. 1)^18^. The researchers replaced the commonly used Terfenol-D with an amorphous FeGaB alloy, which exhibits a greater piezomagnetic coefficient and superior soft magnetic properties. It enables response to weak magnetic fields without the need for bias magnetic fields. The amorphous nature of FeGaB also simplifies the thin-film fabrication process. In addition to the material improvement, the team also optimized the measurement parameters. By carefully choosing detuning the laser frequency, they were able to effectively suppress technical noise in the system to reach the thermal noise limit. In this way, a sensitivity of 1.68 pT Hz^−1/2^ was realized, which constitutes two orders of magnitude improvement over previous mass-producible cavity optomechanical magnetometers^17,18^. The team also demonstrated a proof-of-concept application of detecting pulsed magnetic field signals simulating the corona currents in high-voltage transmission lines.

**Fig. 1:**
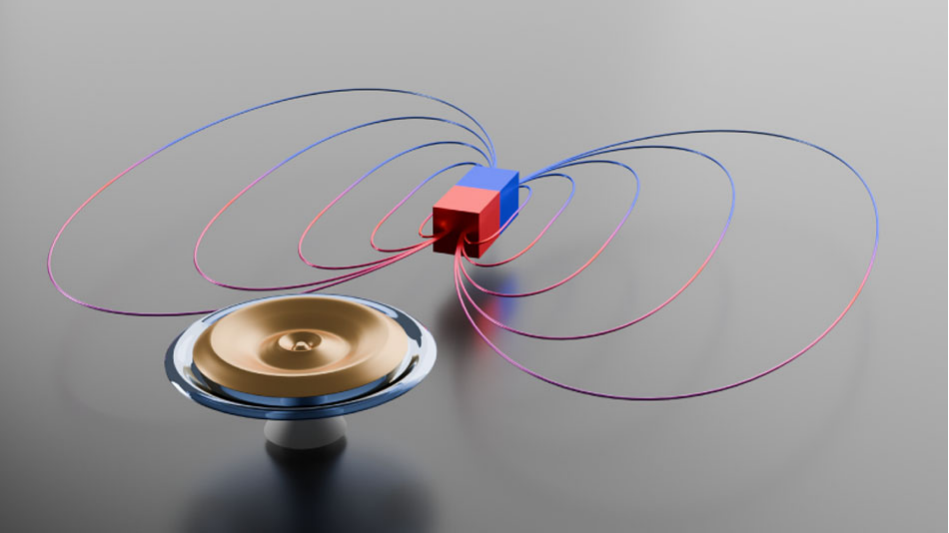
Illustration of the microcavity optomechanical magnetometer using a silica microdisk coated by an FeGaB film

The incorporation of FeGaB material into microcavity optomechanical magnetometers enables the efficient transduction of magnetic signals into measurable mechanical responses. Building upon this work, fully integrated microcavity optomechanical magnetometers can be envisioned by combining FeGaB thin films with CMOS-compatible silicon nitride ring resonators. It paves the way for mass-produced, high-sensitivity integrated magnetometers. These CMOS-compatible magnetometers hold great potential for applications ranging from corona current monitoring to magnetic induction tomography and magnetoencephalography. The convergence of advanced materials and photonic integration in CMOS-compatible platforms is a transformative step towards compact, sensitive, and scalable magnetic sensors.
